# Evaluation of the Xpert Xpress GBS test for rapid detection of group B *Streptococcus* in pregnant women

**DOI:** 10.1128/spectrum.02206-23

**Published:** 2023-12-06

**Authors:** Feiling Wang, Lehui Yi, Fang Ming, Rui Dong, Feng Wang, Ruirui Chen, Xiaoling Hu, Xuri Chen, Bo Sun, Yi-Wei Tang, Yuanfang Zhu, Lijuan Wu

**Affiliations:** 1 Clinical Laboratory, Shenzhen Baoan Women’s and Children’s Hospital, Shenzhen, Guangdong, China; 2 Department of Graduate and Scientific Research, Zunyi Medical University Zhuhai Campus, Zhuhai, Guangdong, China; 3 Department of Obstetrics, Shenzhen Key Medical Discipline, Maternal-Fetal Medicine Institute, Shenzhen Bao'an Women’s and Children’s Hospital, Shenzhen, Guangdong, China; 4 Clinical Affairs, Cepheid, Sunnyvale, California, USA; 5 Medical Affairs, Cepheid, Sunnyvale, California, USA; 6 Danaher Diagnostic Platform China, Shanghai, China; Montefiore Medical Center and Albert Einstein College of Medicine, Bronx, New York, USA

**Keywords:** group B *Streptococcus*, Xpert Xpress GBS, enrichment culture, qPCR, direct culture

## Abstract

Group B *Streptococcus* (GBS) remains the leading cause of serious neonatal infection. Prenatal screening for vaginal and rectal GBS colonization is necessary in late pregnancy. Our objective was to compare the performance of four methods including the Xpert Xpress GBS, standard broth enrichment culture, real-time quantitative PCR (qPCR), and direct culture, for detecting of GBS in rectovaginal specimens. Among 939 pregnant women at 35–37 weeks of gestation recruited in the study, 168 (17.9%) were determined to be GBS-colonized by broth enrichment culture, the gold standard. The sensitivity and specificity of the Xpert Xpress GBS were 94.6% and 95.9%, respectively. The hands-on time and running time of the Xpert Xpress GBS were 1 minute and less than 1 hour, respectively. As a random-access, integrated device, the Xpert Xpress GBS test efficiently and accurately screens rectovaginal swabs for GBS colonization in pregnant women.

**IMPORTANCE:**

This was the first study evaluating the performance of the Xpert Xpress group B *Streptococcus* (GBS) test using rectovaginal swabs from Chinese pregnant women. Compared to the other three assays, the Xpert Xpress GBS test demonstrated high sensitivity and specificity when screening 939 pregnant women for GBS in rectovaginal specimens. Additionally, its reduced time to obtain results makes it valuable for the rapid detection of GBS.

## INTRODUCTION

Group B *Streptococcus* (GBS), also known as *Streptococcus agalactiae*, is a beta-hemolytic gram-positive *Streptococcus*. Approximately 10%**–**30% of pregnant women are colonized with GBS in the vaginal and rectal areas (1, 2). Around 50% of newborns born to mothers colonized with GBS experience vertical transmission, with 1%**–**2% of these infants manifesting invasive disease during early infancy, particularly early-onset GBS disease(3 – 6). A meta-analysis of 135 studies concluded that the overall incidence of invasive GBS disease in infants was 0.49 per 1,000 live births with a case fatality risk of 8.4%. Additionally, the incidence rates of early-onset and late-onset GBS disease were 0.41 and 0.26 per 1,000 live births, respectively (7). To mitigate the risk of perinatal GBS disease, guidelines have been updated by the American College of Obstetricians and Gynecologists (ACOG), the American Academy of Pediatrics (AAP), the American Society for Microbiology (ASM), and the Chinese Expert Consensus on the Prevention of Perinatal GBS Disease(8 – 11). The ACOG guidelines advocate for routine antenatal screening during 36–37 weeks of gestation, employing a GBS prenatal enrichment culture and intrapartum molecular detection. For those identified as GBS-colonized, intravenous antibiotic prophylaxis (IAP) is advised to curtail potential perinatal GBS transmission and subsequent infections (8, 9). Notably, the 2021 Chinese Expert Consensus endorse the recommendation for GBS screening at 35–37 weeks of gestation (11).

A meta-analysis of studies from 37 countries estimated the mean rectovaginal GBS colonization rate in pregnant women to be about 17.9%, with the highest rate in Africa (22.4%), followed by the Americas (19.7%), Europe (19%), and the lowest rate in southeast Asia (11.1%) (3). Hong Kong and Taiwan, which have adopted similar prenatal GBS screening strategies to the United States, reported relatively high GBS colonization rates at 21.8% and 19.6%, respectively (12, 13). In contrast, mainland China had not broadly implemented a GBS screening strategy prior to the introduction of the Chinese Expert Consensus (14, 15). At present, published data indicate that the GBS colonization rates in mainland China are relatively low (16 – 18). A review of 30 studies in mainland China indicated a GBS colonization rate of about 11.3% among pregnant women. Yet, a more comprehensive analysis of 64 studies from mainland China revealed an overall incidence of invasive GBS disease at 0.55 per 1,000 live births, surpassing the global GBS incidence rate of 0.49 per 1,000 live births, and with a case fatality rate of 5% (7, 19). In Shenzhen, China, a metropolitan area near Hong Kong, our laboratory has employed enrichment culture for universal prenatal GBS screening over several years, demonstrating a 14% GBS colonization rate (18).

According to ACOG guidelines, laboratories can utilize nucleic acid amplification testing (NAAT) for GBS screening after an enrichment broth incubation step, achieving a sensitivity similar to conventional culture methods. However, without this enrichment, NAAT’s performance can vary, with failure rates reaching 7%–10% (10). Recently, Cepheid (Sunnyvale, CA, USA) introduced the Xpert Xpress GBS assay, a point-of-care NAAT (POC-NAAT) specifically designed for direct analysis of vaginal/rectal swabs. Its clinical validity remains to be established. Notably, the Xpert Xpress GBS targets two specific GBS genes: one in the coding region for a glycosyl transferase family protein and another linked to a *LysR* family transcriptional regulator of GBS DNA. This contrasts with the earlier Xpert GBS assay, which is based on detecting genomic sequences next to the *cfb* gene (20). To date, neither the Xpert GBS assay nor the Xpert Xpress GBS has been clinically adopted or evaluated in China. However, other prevalent qPCR GBS kits in the region, primarily targeting the *cfb* gene for detection, demonstrate GBS positivity rates of 7.2%–9.2% in direct rectovaginal swab tests (16, 21, 22).

The objective of this study is to evaluate the performance of the Xpert Xpress GBS test for prenatal GBS screening in pregnant women at 35–37 weeks of gestation in China. Rectovaginal swab specimens were collected for GBS testing by the Xpert Xpress GBS, qPCR, and direct culture, with broth enrichment culture as the reference method.

## MATERIALS AND METHODS

### Study design and participants

**Fig 1:**
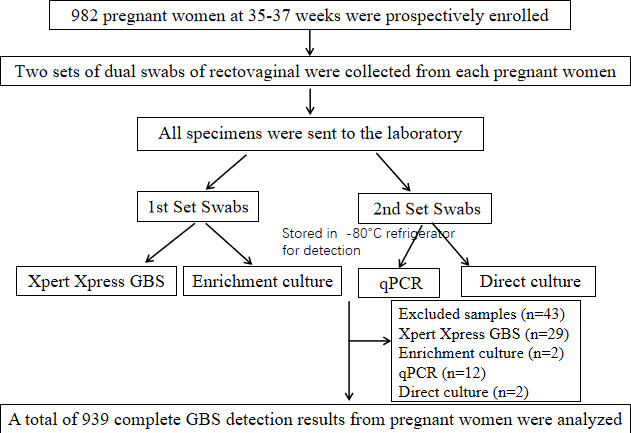
Overall study specimen selection and workflow.

A total of 982 pregnant women, at 35–37 weeks of gestation, attending the obstetrics clinic of Shenzhen Baoan Women’s and Children’s Hospital, were prospectively enrolled from August 20, 2021, to August 18, 2022. Repeat participants were excluded. Rectovaginal sampling was performed by an obstetrician using two sets of dual swabs with transport medium (ESwab, Copan Diagnostics, Brescia, Italy), and all swabs (four swabs per participant) were sent to the laboratory for analysis. Three swabs from each participant were tested by the Xpert Xpress GBS, enrichment culture, and direct culture, respectively. The fourth swab was stored in a −80°C refrigerator for qPCR. We excluded 43 samples due to incomplete testing. Out of these, 29 were from the Xpert Xpress GBS: two due to sample adequacy control (SAC) issues, 21 because of probe check control (PCC) problems, and six due to system component failures. Additionally, two samples were removed from the enrichment culture because of combination errors. Another two samples from direct culture were part of the 12 samples not tested by qPCR due to a swab shortage. Our analysis included 939 pregnant women who had complete results for all four GBS tests (Fig. 1). This study was approved by the Ethics and Research Committee of Shenzhen Baoan Women’s and Children’s Hospital (LLSC-2022–01-04-05-KS).

### Specimen collection

One set of dual swabs was inserted in the lower one-third of the vaginal and rotated for 3**–**5 seconds to collect vaginal secretions without using a speculum. The same dual swabs were then inserted 2 cm beyond the anal sphincter and rotated for another 3**–**5 seconds. The dual swabs were placed in the ESwab transport medium. The sampling process for the second set of dual swabs was repeated. Upon arrival at the laboratory, specimens were processed within 30 minutes for immediate testing using the Xpert Xpress GBS, enrichment culture, and direct culture. Meanwhile, swabs designated for qPCR were stored at −80℃ for subsequent batch testing. Each of the four test methods was conducted only once. The two swabs from the ESwab transport medium were rolled against each other for 5 seconds before testing.

### Xpert Xpress GBS

The Xpert Xpress GBS test (Cepheid, Sunnyvale, CA, USA), performed on the GeneXpert Instrument Systems, is an automated qualitative test for the detection of two GBS chromosomal targets using real-time PCR. The test uses proprietary predetermined cycle threshold (*C_T_*) thresholds and cutoffs to autonomously categorize results as GBS-positive or GBS-negative based on GBS target DNA detection. Three quality controls are integrated into each test: sample processing control (SPC), SAC, and PCC. Single-use disposable cartridges, housing the PCR process and containing the necessary PCR reagents, are essential for the test. The swab was inserted into the Xpert Xpress GBS cartridge’s sample chamber, with the shaft subsequently broken as per the manufacturer’s instructions. After sealing the cartridge, it was positioned in the GeneXpert instrument for automated processing, amplification, and detection. Hands-on-time (HOT) was noted from the moment the swab was placed in the cartridge to its insertion into the GeneXpert. The running time (RT) captured the duration of the GeneXpert instrument’s test.

### qPCR

All reagents were sourced from the GBS Nucleic Acid Detection Kit by Tianlong (Suzhou, China), a proprietary kit certified by the Chinese FDA. This kit features a pair of GBS-specific primers for the *cfb* gene, a TaqMan probe for PCR, and an internal control to detect amplification inhibition during DNA extraction. It also provides both positive and negative controls. The DNA extraction and PCR process were conducted according to the manufacturer’s instructions. The swabs in the transport medium, previously frozen at −80℃ for qPCR testing, were thawed at room temperature for DNA extraction. Each swab was transferred into an Eppenddorf (EP) tube containing 1 mL of sterile saline and mixed thoroughly. The EP tube was then centrifuged at 12,000 rpm for 5 minutes and the supernatant removed. Fifty microliters of nucleic acid extraction reagent was added to the pellet, and the EP tube was then heated at 100°C for 10 minutes. After another centrifugation at 12,000 rpm for 5 minutes, 4 µL of the resulting supernatant was combined with 36 µL of amplification reagent to prepare a 40 µL PCR reaction mix. Amplification was performed on the ABI 7500 real-time PCR System (Life Technologies, Singapore) using the following conditions: one cycle at 95°C for 3 minutes, followed by 40 cycles at 94°C for 15 seconds and 60°C for 30 seconds. Samples were considered positive with the cycle threshold value set at *C_T_* ≤ 38. The HOT for qPCR represented the duration needed for manual nucleic acid extraction from batched samples and the manual procedures during reagent preparation. The RT for qPCR covered the centrifugation, heat treatment, and amplification processes on the ABI 7500.

### Enrichment culture and direct culture

One swab was transferred into a Lim broth tube (Kailin, Jiangmen, China). The swab was rotated and rinsed in the liquid along the sides of the tube several times and snapped at the score line into the tube. The Lim broth tube was placed in a 37°C, 5% CO_2_ incubator overnight and the contents sub-cultured to a 5% sheep blood agar plate (Dijing, Guangzhou, China), which was incubated at 37°C, 5% CO_2_ for 18–24 hours. Direct culture was performed by inoculating a swab onto 5% sheep blood agar and incubating the plate overnight at 37°C, 5% CO_2_ for 18–24 hours. All colonies displaying either beta-hemolytic or non-hemolytic characteristics suspicious of GBS on blood agar plates were subjected to identification using MALDI-TOF mass spectrometry (MALDI-TOF-MS) (Bruker Daltonik, Germany).

Capsular serotyping and CAMP factor detection were performed on nine isolates using the latex agglutination test (Reference 23) and the Christie-Atkins-Munch-Petersen (CAMP) test (Reference 24), following the established procedures outlined in these references (23, 24). The HOT for enrichment culture was defined as the time taken to inoculate Lim broth with the specimen, use of sterile cotton swabs to inoculate blood agar plates with the broth culture, visual inspection of agar plates for characteristic colonies, and manual preparation of isolates for identification by MALDI-TOF-MS. The turnaround time (TAT) for enrichment culture was measured from the initial sample collection to the reporting of results.

### Statistical method

The sensitivity and specificity of the Xpert Xpress GBS, qPCR, and direct culture were determined using a 2 × 2 contingency table, with enrichment culture serving as the gold standard. The median *C_T_* values for both the Xpert Xpress GBS and qPCR assays were recorded, and their differences were evaluated using non-parametric tests. The McNemar’s test was used to compare performance differences between the Xpert Xpress GBS and qPCR, with *P* values less than 0.05 considered statistically significant. Statistical analyses were performed using SPSS Statistics 22.0 (IBM, Armonk, NY, USA).

## RESULTS

### Demographics

The 939 participants were grouped by age as follows: 18**–**24 years (*n* = 58), 25**–**34 years (*n* = 741), and 35**–**44 years (*n* = 140). The detailed age distribution can be found in Table S1. Notably, out of the 939 participants, four pregnant women had consumed antibiotics within 2 weeks prior to the GBS screening.

### Performance of the Xpert Xpress GBS, qPCR and direct culture compared to enrichment culture

A total of 939 rectovaginal specimens were tested using four methods to detect the presence of GBS. Compared to enrichment culture, the sensitivity and specificity of the Xpert Xpress GBS were 94.6% and 95.9%, respectively. For qPCR, these values were 78.6% and 96%, and for direct culture, they were 72% and 99.6% (Table 1). There was a significant difference in sensitivity between the Xpert Xpress GBS and qPCR through McNemar’s test (*P* = 0.000) (Table 1).

**TABLE 1:** Performance of the Xpert Xpress GBS, qPCR, and direct culture assays in comparison with enrichment culture

Assay	Assay/referee^*b*^				Sensitivity^*a*^ (%) (95% CI)	Specificity (%) (95% CI)	PPV (%) (95% CI)	NPV (%) (95% CI)
	+/+	+/−	−/+	−/−				
Xpert Xpress GBS	159	32	9	739	94.6 (90.1–97.2)	95.9 (94.2–97.0)	83.3 (77.3–87.9)	98.8 (97.7–99.4)
qPCR	132	31	36	740	78.6 (71.8–84.1)	96.0 (94.4–97.2)	81.0 (74.3–86.3)	95.4 (93.6–96.6)
Direct culture	121	3	47	768	72.0 (64.8–78.3)	99.6 (98.9–99.9)	97.6 (93.1–99.2)	94.2 (92.4–95.6)

^*a*^ The sensitivity of the Xpert Xpress GBS and qPCR compared by McNemar test, *P* = 0.000.

^*b*^ Referee, enrichment culture results; +, positive; −, negative; PPV, positive predictive value; NPV, negative predictive value.

Out of 939 evaluated GBS test results, 20.3% (191/939) was positive by the Xpert Xpress GBS, 17.4% (163/939) by qPCR, 17.9% (168/939) by enrichment culture, and 13.2% by direct culture (Table S2). A total of 132 enrichment culture-positive specimens (78.6%, 132/168) were also positive by both the Xpert Xpress GBS and qPCR, and the median *C_T_* value for the Xpert Xpress GBS was 27.3, which was significantly lower than the qPCR’s median *C_T_* value of 30.5, based on a non-parametric test (*P* = 0.000). The median *C_T_* values for the eight specimens that were GBS-positive by both the Xpert Xpress GBS and qPCR but enrichment culture-negative were 33.6 and 35.9, respectively. Both the Xpert Xpress GBS and qPCR were negative for nine specimens that were enrichment culture-positive. There were 74 specimens with divergent results between the Xpert Xpress GBS and qPCR. Specifically, 24 of these were exclusively positive by the Xpert Xpress GBS, having a median *C_T_* value of 39.40 (Table 2).

**TABLE 2:** Comparison of the Xpert Xpress GBS, qPCR, and enrichment culture results (*N* = 939)^*b*^

No. of specimens	Xpert Xpress GBS (median *C_T_* value)	Enrichment culture	qPCR (median *C_T_* value)
132	+ (27.3) **^*a*^**	+	+ (30.5) **^*a*^**
27	+ (35.9)	+	−
9	−	+	−
8	+ (33.6)	−	+ (35.9)
24	+ (39.4)	−	−
23	−	−	+ (36.2)
716	−	−	−
Positivity rate (%)	20.3 (191/939)	17.9 (168/939)	17.4 (163/939)

^*a*^ Difference in *C_T_* values between the Xpert Xpress GBS and qPCR (in 132 positive samples) was analyzed using a non-parametric test (*P* = 0.000).

^*b*^ +, positive; −, negative.

## DISCUSSION

In this study, for the first time, we evaluated the Xpert Xpress GBS’s ability to directly detect GBS from rectovaginal swabs. Impressively, the test showed a sensitivity of 94.6%, surpassing both qPCR and direct culture methods. While its specificity was consistent with qPCR, it is slightly lower than direct culture. The enhanced sensitivity of the Xpert Xpress can likely be attributed to its innovative dual-target feature and its refined limit of detection (LOD). Cepheid, the manufacturer, has indicated that the Xpert Xpress GBS boasts a LOD ranging from 3 to 51 CFU/Swab. This is more sensitive than the previous Xpert GBS with an LOD of 250 CFU/Swab. Furthermore, this test can identify up to 10 GBS serotypes, ranging from Ia to X. In this study, the Xpert Xpress documented a GBS positivity rate of 20.3%, the highest among all four methods we analyzed. Specifically, it identified an extra 32 cases in contrast to the enrichment culture: 24 of these cases presented with a median *C_T_* value of 39.4, and qPCR detected the remaining 8. It is possible that GBS isolates could not be recovered from these samples due to the presence of non-viable organisms in enrichment culture. However, because additional testing was not performed to confirm the 24 extra GBS detections by the Xpert Xpress GBS assay, we cannot exclude the possibility that these detections represent false-positive results. Among the 168 samples identified as GBS-positive by enrichment culture, the Xpert Xpress failed to detect GBS in nine cases. Each of these nine isolates showed beta-hemolysis and tested positive on the CAMP assay, with serotype distribution as follows: five serotype III, two serotype Ia, one serotype Ib, and one serotype V. Notably, none of the undetected cases corresponded to the rarer capsular serotypes VI through X. This suggests that the missed detections by the Xpert Xpress may be attributed to a lower bacterial load in the samples.

For investigation of the discrepant results for the Xpert Xpress versus the qPCR, analyzing the positive cases identified by each method, the Xpert Xpress test exhibited a broader range of *C_T_* values, from 19.1 to 43.1, in contrast to qPCR, which showed a range from 21.0 to 37.9. Among the 132 cases positive by both methods, the *C_T_* values from the Xpert Xpress were significantly lower than those from qPCR, suggesting a higher efficiency of the GeneXpert system’s automated DNA extraction over the manual process required for qPCR. Notably, the observed discrepancies between the Xpert Xpress test and the qPCR method could be due to differences in swab sets used for each test. The Xpert Xpress uniquely identified 27 cases not detected by qPCR, whereas qPCR identified 23 cases not detected by the Xpert Xpress.

To evaluate the processing and detection times of the Xpert Xpress GBS, qPCR, and enrichment culture, we assessed the average HOT and RT for each method. The Xpert Xpress GBS required an average HOT of 1 minute and had an RT of 43 minutes per test. For qPCR, when analyzing a batch of 94 specimens, the average HOT was approximately 60 minutes, with an RT of 70 minutes. Enrichment culture had an average HOT of 8 minutes, while the TAT was 48.5 ± 4.5 hours per specimen (Table S3).

In this study, the Xpert Xpress assay reported 21 PCC issues and six system component failures from 982 samples, suggesting that enhancements to the testing system are necessary. Additionally, the assay halted two tests due to SAC issues, which could be advantageous for monitoring sample quality.

This study presents several limitations. Firstly, the ACOG and ASM guidelines recommend the use of enrichment culture or enrichment-based NAAT for antepartum GBS screening and reserve POC-NAAT or NAAT for intrapartum use. This study was only conducted in antepartum women and did not assess during the intrapartum period. Future research should focus on evaluating the performance in intrapartum conditions. Secondly, as this was a single-center study involving Chinese pregnant women, it lacks validation across multiple centers. Lastly, due to the different GBS screening timeline recommended by the Chinese Expert Consensus compared to the ACOG, only 53 out of the 939 pregnant women in this study were at 36–37 weeks of gestation.

In conclusion, our study uncovers that the rate of GBS colonization in mainland China is markedly higher than the previously reported data, which varied from 3.7% to 14.5% (25). This aligns with the meta-analyses results worldwide (3, 26). The Xpert Xpress GBS assay demonstrated high sensitivity and specificity, coupled with a rapid processing time, which are significant advantages. However, additional evaluations under varied conditions are needed to provide a comprehensive assessment of its performance.

## SUPPLEMENTAL MATERIAL

Table S1 spectrum.02206-23-s0001.docx Age distribution of participants.

Table S2 spectrum.02206-23-s0002.doc Detailed GBS results and positivity rates of the four assays.

Table S3 spectrum.02206-23-s0003.docx Comparison of workflows of Xpert Xpress GBS, enrichment culture, and qPCR assays.

ASM does not own the copyrights to Supplemental Material that may be linked to, or accessed through, an article. The authors have granted ASM a non-exclusive, world-wide license to publish the Supplemental Material files. Please contact the corresponding author directly for reuse.
