# Supplementary material for: Evaluation of the Xpert Xpress GBS test for rapid detection of group B Streptococcus in pregnant women
Source: Microbiol Spectr. 2023 Dec 6;12(1):e02206-23. doi: 10.1128/spectrum.02206-23 (PMC10783076; doi:10.1128/spectrum.02206-23)
Supplement: Table S1 — Age distribution of participants. [file spectrum.02206-23-s0001.docx]

**Supplemental Table 1** Age distribution of participants

| **Age(yrs)** | **No. of participants** | **%** |
| --- | --- | --- |
| 18-24 | 58 | 6.2 |
| 25-34 | 741 | 78.9 |
| 35-44 | 140 | 14.9 |
| Total | 939 | 100.0 |
