# Supplementary material for: Evaluation of the Xpert Xpress GBS test for rapid detection of group B Streptococcus in pregnant women
Source: Microbiol Spectr. 2023 Dec 6;12(1):e02206-23. doi: 10.1128/spectrum.02206-23 (PMC10783076; doi:10.1128/spectrum.02206-23)
Supplement: Table S2 — Detailed GBS results and positivity rates of the four assays. [file spectrum.02206-23-s0002.doc]

**Supplemental Table 2 Distribution of GBS results by four methods (N = 939)**

| **No. of specimens** | **Enrichment culture** | **Direct culture** | **Xpert Xpress GBS** | **qPCR** |
| --- | --- | --- | --- | --- |
| 108 | **+** | **+** | **+** | **+** |
| 11 | **+** | **+** | **+** | **-** |
| 2 | **+** | **+** | **-** | **-** |
| 24 | **+** | **-** | **+** | **+** |
| 16 | **+** | **-** | **+** | **-** |
| 7 | **+** | **-** | **-** | **-** |
| 1 | **-** | **+** | **+** | **+** |
| 1 | **-** | **+** | **+** | **-** |
| 1 | **-** | **+** | **-** | **+** |
| 7 | **-** | **-** | **+** | **+** |
| 23 | **-** | **-** | **+** | **-** |
| 22 | **-** | **-** | **-** | **+** |
| 716 | **-** | **-** | **-** | **-** |
| Positivity rate | 17.9% (168/939) | 13.2% (124/939) | 20.3% (191/939) | 17.4% (163/939) |

+, positive; −, negative.

This table showed the detailed GBS results and positivity rates of these four assays.
