# Supplementary material for: Evaluation of the Xpert Xpress GBS test for rapid detection of group B Streptococcus in pregnant women
Source: Microbiol Spectr. 2023 Dec 6;12(1):e02206-23. doi: 10.1128/spectrum.02206-23 (PMC10783076; doi:10.1128/spectrum.02206-23)
Supplement: Table S3 — Comparison of workflows of Xpert Xpress GBS, enrichment culture, and qPCR assays. [file spectrum.02206-23-s0003.docx]

**Supplemental Table 3** Comparison of workflows of Xpert Xpress GBS, Enrichment culture and qPCR assays

| Assay | Specimen  capacity | Hands-on time (minutes) | Running time (minutes)* | Turnaround time (hours) |
| --- | --- | --- | --- | --- |
| Xpert Xpress GBS ^a^ | 1-32 | 1 | 43 | N.A. |
| Enrichment culture ^a^ | N.A. | 8 | N.A. | 48.5±4.5 |
| qPCR ^b^ | 94 | 60 | 70 | N.A. |

N.A., not available. Hands on time, time for manual sample handling; turnaround time, time from sample to result reporting.

^*^Running time was recorded in Xpert Xpress GBS as the time the sample was tested on the instrument, and in qPCR as the time of centrifugation, warm bath, amplification by ABI 7500.

^a^The detection time of a single specimen.

^b^The detection time of a single batch of 94 specimens.
